# REVEL and BayesDel outperform other *in silico* meta-predictors for clinical variant classification

**DOI:** 10.1038/s41598-019-49224-8

**Published:** 2019-09-04

**Authors:** Yuan Tian, Tina Pesaran, Adam Chamberlin, R. Bryn Fenwick, Shuwei Li, Chia-Ling Gau, Elizabeth C. Chao, Hsiao-Mei Lu, Mary Helen Black, Dajun Qian

**Affiliations:** 1 Ambry Genetics, 15 Argonaut, Aliso Viejo, CA 92656 USA; 2 Divsion of Genetics and Genomics, University of California, Irvine, CA 92697 USA

**Keywords:** Statistical methods, Genetic testing

## Abstract

Many *in silico* predictors of genetic variant pathogenicity have been previously developed, but there is currently no standard application of these algorithms for variant assessment. Using 4,094 ClinVar-curated missense variants in clinically actionable genes, we evaluated the accuracy and yield of benign and deleterious evidence in 5 *in silico* meta-predictors, as well as agreement of SIFT and PolyPhen2, and report the derived thresholds for the best performing predictor(s). REVEL and BayesDel outperformed all other meta-predictors (CADD, MetaSVM, Eigen), with higher positive predictive value, comparable negative predictive value, higher yield, and greater overall prediction performance. Agreement of SIFT and PolyPhen2 resulted in slightly higher yield but lower overall prediction performance than REVEL or BayesDel. Our results support the use of gene-level rather than generalized thresholds, when gene-level thresholds can be estimated. Our results also support the use of 2-sided thresholds, which allow for uncertainty, rather than a single, binary cut-point for assigning benign and deleterious evidence. The gene-level 2-sided thresholds we derived for REVEL or BayesDel can be used to assess *in silico* evidence for missense variants in accordance with current classification guidelines.

## Introduction

*In silico* prediction of variant pathogenicity is one of the eight evidence categories recommended by the American College of Medical Genetics and American College of Pathologist (ACMG/AMP) guidelines^1^. Many *in silico* algorithms have been developed to predict the degree of sequence conservation and functional impact of missense variants, each of which generates a score based on tolerance to variation. Assessment of *in silico* evidence based on the observed concordance of multiple scores yields a high rate of discordant predictions, even among well-classified variants, suggesting that such an approach is not ideal^2,3^. Recently, several *in silico* meta-predictors have been developed from analyses of multiple individual scores, and have demonstrated superior performance to that of individual predictors, although robust, systematic comparison of these approaches is lacking^4–6^.

Furthermore, *in silico* meta-predictors have primarily been trained on large numbers of variants selected from genome-wide data. Yet, variants from same gene share common features and gene-level transcription facilitates the events that drive the development and progression of disease, as well as response to therapy^7^. Global applications of *in silico* scores may result in a large number of false predictions as a one-size-fits-all approach^8,9^, but gene-level thresholds for assessing benign and deleterious evidence are unavailable, in practice. In addition, while many algorithms recommend binary thresholds for assessing *in silico* evidence, clinical recommendations based on thresholds for “likely benign” and “likely pathogenic” are 2-sided^10^.

Given the wide variety of *in silico* algorithms and approaches available, there is currently no standard application of these tools for variant assessment; many laboratories use different tools and thresholds for evidence of pathogenic or benign classification^11,12^. As such, the 2015 ACMG/AMP guidelines assign only a supporting level of evidence to *in silico* predictions^1^. In this study, we aimed to assess *in silico* evidence for pathogenicity of missense variants using gene-level and generalized (all genes combined) 2-sided thresholds, compare prediction performance among 5 commonly used meta-predictors, and identify the thresholds corresponding to the meta-predictor(s) with the best overall prediction performance that can be used to assess *in silico* evidence in accordance with the ACMG/AMP guidelines.

## Methods

### Data

To evaluate the prediction performance of various *in silico* scores, we compiled a dataset of 4,094 classified missense variants in 66 clinically relevant genes from ClinVar. Variants were included if reviewed by expert panel or classified by any of 6 submitters that consistently provide assertion criteria: Ambry Genetics, Emory Genetics Laboratory, GeneDx, InSiGHT, InVitae and Sharing Clinical Reports Project. For each variant, we defined its consensus class as the most supported category among benign/likely benign (B/LB), pathogenic/likely pathogenic (P/LP) and variants of uncertain significance (VUS), i.e. pathogenic/likely pathogenic if *N*_*P/LP*_ ≥ max(*N*_*VUS*_, 1) and *N*_*B/LB*_ = 0 or benign/likely benign if *N*_*B/LB*_ ≥ max(*N*_*VUS*_, 1) and *N*_*P/LP*_ = 0. Variants with conflicting classes (i.e., *N*_*P/LP*_ ≥ 1 and *N*_*B/LB*_ ≥ 1) or classified as VUS by the majority of submitters (i.e., *N*_*VUS*_ > max(*N*_*B/LB*_, *N*_*P/LP*_)) were excluded from analysis.

*In silico* scores were obtained for 5 meta-predictors (CADD^4^, MetaSVM^5^, Eigen^13^, REVEL^14^ and BayesDel^6^) and 2 individual predictors (SIFT^15^ and PolyPhen2^16^) from the dbNSFP v3.5c database (August 6, 2017)^17^ and related websites (Supplementary Table S1). Datasets included classified variants in 66 genes (Supplementary Table S2) and a subset of variants in 20 genes, each containing at least 10 B/LB and 10 P/LP variants. Approximately 5.1%, 0.6%, 0.1%, and 2.1% of SIFT, PolyPhen2, MetaSVM, and Eigen values, respectively, were missing across the 4,094 variants. CADD, REVEL, and BayesDel had no missing values (Supplementary Table S3).

### Gene-level and generalized thresholds

To derive gene-level thresholds for the 20 genes with ≥10 B/LB and ≥10 P/LP variants, we fit models using Firth logistic regression^18^ for variants within each gene. Firth logistic regression was used to ensure model robustness under the scenarios of separation or sparse benign/likely benign and pathogenic/likely pathogenic outcomes^19,20^. Gene-level 2-sided thresholds for *in silico* evidence were derived at the predicted probabilities 0.2 and 0.8, respectively. These predicted probabilities were projected from the regression model, depend in part on the proportion of pathogenic variants in the dataset constructed, and were not based on estimation in any patient population; the probabilities were chosen to achieve ≥90% overall predictive accuracy for most meta-predictors. Thus, *in silico* data was assessed for benign evidence, deleterious evidence or no evidence using gene-level thresholds for the 20 genes. For comparison of gene-level thresholds to those estimated with a broader approach aggregating variation across all genes, we further estimated generalized thresholds for the 20 genes using Firth logistic regression and predicted probabilities, as outlined above. Moreover, there were 46 genes for which gene-level thresholds were not estimable, primarily due to lack of sufficient numbers of B/LB or P/LP classified variants for analysis. As an exploratory analysis, we further derived generalized thresholds for the combined set of 66 genes, using the methods described above. For all analyses, variants with missing scores were assigned no evidence for the corresponding *in silico* predictor. To assess the robustness of the gene-level thresholds we derived for benign and deleterious evidence, we estimated 90% confidence intervals (CI) from 10,000 bootstrapping replicates stratified by classification status.

### Cross-validation and sensitivity analysis

To account for potential overfitting, the predictive performance of each meta-predictor was evaluated using leave-one-out cross-validation, in which the assigned evidence was compared to the B/LB and P/LP status from ClinVar consensus classification. Performance statistics were evaluated using positive predictive value (PPV), negative predictive value (NPV) and yield rate (YR). YR was defined as the proportion of variants received benign or deleterious evidence among all evaluated variants. To assess the joint performance of accuracy and yield, we computed overall prediction performance (OPP), defined as the root mean square of PPV, NPV and YR, i.e., OPP = $\sqrt{(PP{V}^{2}+NP{V}^{2}+Y{R}^{2})/3}$. Differences in PPV, NPV, or YR between predictors were each tested by Fisher’s exact test. Differences in OPP were tested by a Monte Carlo permutation test with 10,000 permutations that each randomly exchanged all assigned evidence among comparator predictors. Performance statistics for gene-level and generalized thresholds using 20 genes are shown in Table 1. For the meta-predictor demonstrating the highest OPP statistic, we report the optimized gene-level thresholds in Table 2. As an exploratory analysis, performance statistics for generalized thresholds using all 66 genes were also compared to those obtained using a combination of gene-level and generalized thresholds, and are provided in Supplementary Table S5.

**Table 1:** Prediction performance of *in silico* evidence assignment (2,153 variants in 20 genes).

Method	Performance statistic (Rank)^a^								
	TP	TN	FP	FN	NE	PPV	NPV	YR	OPP
SIFT/PolyPhen2 agreement	888	649	177	54	385	0.834 (11)	0.923 (9)	0.821 (1)	0.861 (10)
Gene-level thresholds									
CADD	746	707	60	38	602	0.926 (7)	0.949 (7)	0.720 (9)	0.871 (8)
MetaSVM	848	746	57	39	463	0.937 (5)	0.950 (6)	0.785 (4)	0.894 (4)
Eigen	850	761	51	27	464	0.943 (3)	0.966 (2)	0.784 (5)	0.901 (3)
REVEL	858	784	40	33	438	0.955 (2)	0.960 (3)	0.797 (3)	0.907 (2)
BayesDel	859	798	39	40	417	0.957 (1)	0.952 (5)	0.806 (2)	0.908 (1)
Generalized thresholds									
CADD	563	525	75	18	972	0.882 (10)	0.967 (1)	0.549 (11)	0.819 (11)
MetaSVM	848	697	75	64	469	0.919 (8)	0.916 (11)	0.782 (6)	0.875 (6)
Eigen	747	684	80	31	611	0.903 (9)	0.957 (4)	0.716 (10)	0.865 (9)
REVEL	846	673	52	60	522	0.942 (4)	0.918 (10)	0.758 (7)	0.876 (5)
BayesDel	825	672	58	52	546	0.934 (6)	0.928 (8)	0.746 (8)	0.874 (7)

^a^All performance statistics, except those for SIFT/PolyPhen2 agreement, were evaluated by leave-one-out cross-validation. Ranks in parentheses were the descending orders of performance statistics among comparison methods. The p-values of Monte Carlo permutation tests for differences of OPP statistics between evidence of gene-level versus generalized thresholds were 0.0005, 0.09, 0.002, 0.006 and 0.003 for CADD (OPP: 0.871 vs. 0.819), MetaSVM (OPP: 0.894 vs. 0.875), Eigen (OPP: 0.901 vs. 0.865), REVEL (OPP: 0.907 vs. 0.876) and BayesDel (OPP: 0.908 vs. 0.874), respectively. TN, true negative; FN, false negative; TP, true positive; FP, false positive; NE, no evidence; PPV, positive predictive value; NPV, negative predictive value; YR, yield rate; OPP, overall prediction performance.

**Table 2:** Gene-level thresholds for assigning benign and deleterious *in silico* evidence in missense variants^a^.

Gene	Thresholds of REVEL scores		Thresholds of BayesDel scores	
	T_BE_	T_DE_	T_BE_	T_DE_
*ATM*	0.359	0.689	−0.180	0.216
*ATP7B*	0.514	0.731	−0.076	0.248
*BRCA1*	0.628	0.824	0.147	0.425
*BRCA2*	0.581	0.974	0.080	0.500
*CFTR*	0.438	0.727	−0.032	0.277
*COL3A1*	0.515	0.762	0.026	0.329
*FBN1*	0.326	0.597	−0.328	0.047
*KCNH2*	0.417	0.649	−0.176	0.127
*MLH1*	0.109	0.815	0.107	0.423
*MSH2*	0.562	0.862	0.085	0.426
*MSH6*	0.556	0.881	0.095	0.419
*MUTYH*	0.214	0.661	−0.078	0.263
*MYBPC3*	0.013	0.511	−0.531	0.012
*NF1*	0.261	0.605	−0.191	0.077
*NSD1*	0.400	0.705	−0.082	0.268
*RET*	0.481	0.732	−0.122	0.300
*RYR2*	0.349	0.597	−0.233	0.038
*SCN5A*	0.425	0.704	−0.108	0.180
*TP53*	0.536	0.667	−0.003	0.132
*TSC2*	0.703	0.970	0.244	0.561

^a^The 2-sided thresholds, denoted as T_BE_ and T_DE_, are the lower and upper limits of REVEL or BayesDel scores for assigning BE and DE, respectively. Gene-level thresholds for BE and DE were estimated at probabilities of pathogenicity 0.2 and 0.8, respectively. BE, benign evidence; DE, deleterious evidence.

In an effort to minimize the overlap of variants included in this analysis and those used to train any of the 5 meta-predictor models, we also performed a sensitivity analysis in 20 genes to assess meta-predictor performance among only those variants evaluated by all submitters in ClinVar during the period from September 2015 to August 2017.

### Alternative methods

For comparison, we evaluated the performance of *in silico* prediction using generalized thresholds from a combination of 20 genes and that of SIFT/PolyPhen2 agreement (Table 1). Evidence for SIFT/PolyPhen2 agreement was assessed as deleterious if SIFT < 0.05 and PolyPhen2 = “possibly/probably damaging”, or benign if SIFT ≥ 0.05 and PolyPhen2 = “benign”. All analyses were conducted with R for Statistical Computing version 3.3.3.

## Results

### Evidence assignment using gene-level and generalized thresholds

**Figure 1:**
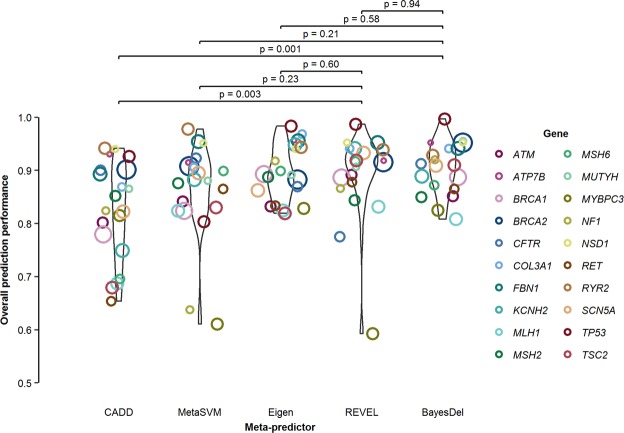
Assessment of *in silico* evidence in missense variants. The OPP statistics were reported in each of the 20 genes using gene-level thresholds. The OPP in 20 genes combined were 0.871, 0.894, 0.901, 0.907 and 0.908 for CADD, MetaSVM, Eigen, REVEL and BayesDel, respectively. P-values for pairwise comparisons were each estimated from Monte Carlo permutation test with 10,000 permutations. OPP, overall prediction performance.

Using gene-level thresholds, REVEL and BayesDel achieved the highest OPP, 0.907 and 0.908, respectively (Table 1). Compared to CADD, MetaSVM, and Eigen, predictions using REVEL had equivalent NPV (−0.6% to 1.1% relative change, all *p* > 0.33) and improved PPV (1.3% to 3.2% higher, all *p* < 0.28), YR (1.5% to 10.6% higher, all *p* < 0.37) and OPP (0.6% to 4.1% higher, all *p* < 0.60) (Table 1; Fig. 1). Like REVEL, BayesDel outperformed CADD, MetaSVM, and Eigen. REVEL and BayesDel had nearly equivalent PPV, NPV, YR and OPP (all *p* > 0.44 for differences between the two). For each of 5 meta-predictors in 20 genes, evidence assignment using gene-level thresholds consistently outperformed that of generalized thresholds (2.2% to 5.9% higher OPP, all *p* < 0.09) (Table 1). While REVEL and BayesDel were concordant for 86.4% of variants and similarly outperformed the other meta-predictors, they did not agree on assigned evidence for 13.6% of variants; 6.6% were better predicted with REVEL and 7.0% with BayesDel (Supplementary Table S4).

Similar trends in prediction performance were observed for thresholds estimated across 66 genes compared to the 20-gene analysis. Using a combination of gene-level and generalized thresholds, REVEL and BayesDel achieved higher OPP than those of CADD, MetaSVM and Eigen (0.8% to 9.7% higher, all *p* < 0.32) (Supplementary Table S5). REVEL and BayesDel were discordant on assigned evidence for 14.5% of variants, roughly half of which were better predicted with REVEL and half with BayesDel (Supplementary Table S6).

### Evidence assignment by alternative methods

Our 20-gene analysis also demonstrated that evidence assigned using REVEL or BayesDel gene-level and generalized thresholds each had consistently better prediction performance than those derived using SIFT/PolyPhen2 agreement (Table 1). Evidence assigned using REVEL vs. SIFT/PolyPhen2 agreement was concordant for 1,463 (68.0%) variants (Supplementary Table S7). Among the 690 variants for which evidence assignment was discordant, use of thresholds based on SIFT/PolyPhen2 agreement resulted in 27.5% false positives/negatives, in contrast to REVEL’s 4.6% false positives/negatives. Proportions of true and false predictions similarly favored BayesDel over SIFT/PolyPhen2 agreement (Supplementary Table S8). Using REVEL or BayesDel thresholds achieved 4.9% or 5.6% more correct predictions than SIFT/PolyPhen2, respectively (Supplementary Tables S7 and S8). Comparisons of REVEL or BayesDel with SIFT/PolyPhen2 agreement for all 66 genes yielded similar results (Supplemental Tables S9 and S10). Using REVEL or BayesDel thresholds yielded 12.4% or 11.7% more correct predictions than SIFT/PolyPhen2, respectively.

### Sensitivity analysis

To assess the impact of classified variants that may overlap between meta-predictor training datasets and our evaluation dataset, we validated the prediction performance of 5 meta-predictors in a subset of 870 missense variants in 20 genes that were unlikely to have been previously used in the training datasets of meta-predictors. Similar to the results observed in the full dataset, REVEL and BayesDel had nearly identical performance statistics, and both had higher OPP than CADD, MetaSVM and Eigen (0.8% to 5.6% higher, all *p* < 0.68) (Supplementary Table S11).

### Thresholds for assigning *in silico* evidence

**Figure 2:**
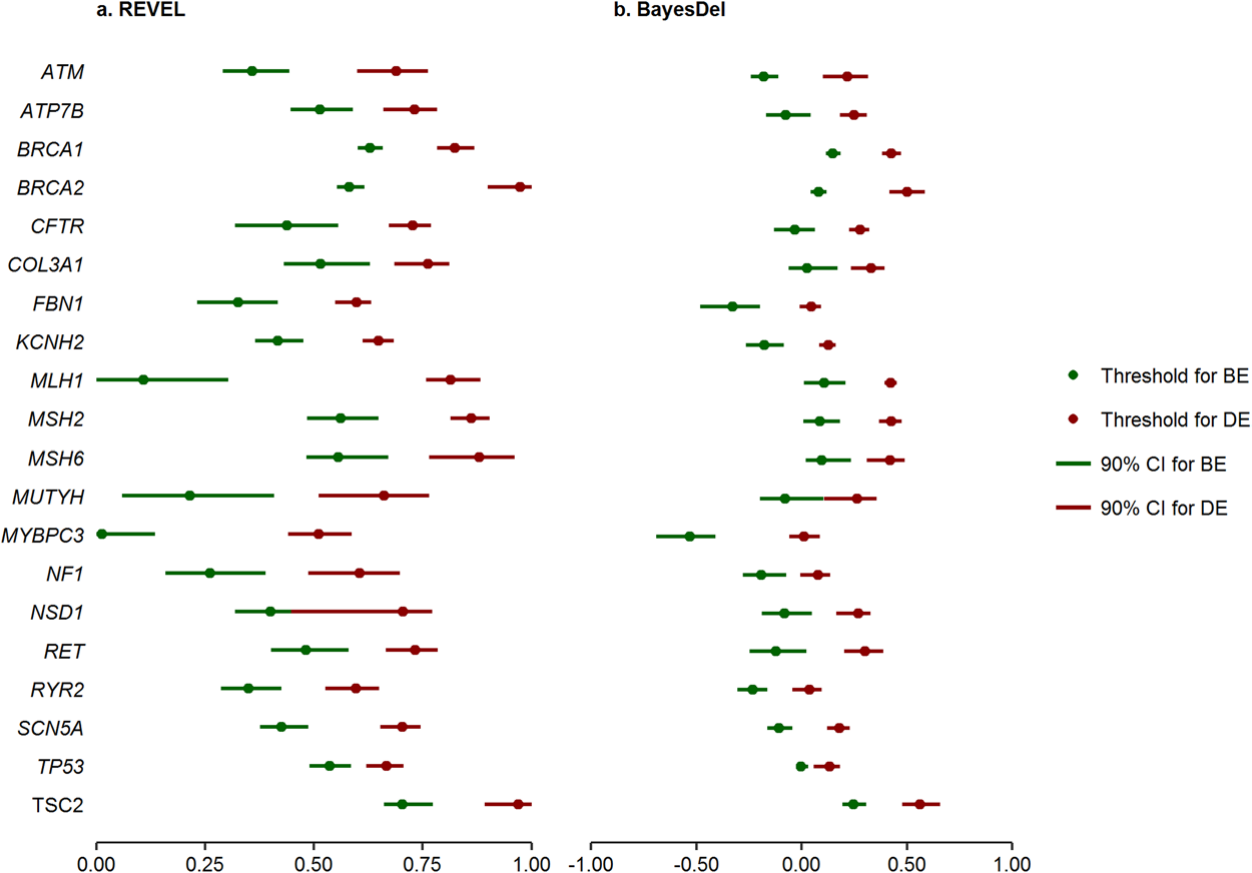
Variation in thresholds for assigning benign and deleterious *in silico* evidence across 20 genes. (**a**) Gene-level 2-sided thresholds and their 90% confidence intervals (CI) for REVEL. (**b**) Gene-level 2-sided thresholds and their 90% confidence intervals (CI) for BayesDel. Thresholds for BE and DE were represented by green and red dots, respectively. BE, benign evidence; DE, deleterious evidence.

Gene-level 2-sided thresholds of REVEL and BayesDel scores for assessment of benign and deleterious *in silico* evidence in missense variants are provided in Table 2. A single, binary cut-point of 0.5 and 0 is often used for REVEL and BayesDel, respectively, to distinguish pathogenic evidence from benign^2^. However, our derived thresholds for benign evidence were above these cut-points for several genes (Fig. 2). For both REVEL and BayesDel, there was substantial variation among thresholds across genes, highlighting the importance of using gene-level thresholds whenever possible. In addition, the 90% CI for gene-level benign and deleterious thresholds were non-overlapping in all except one gene (*NSD1*, REVEL-based thresholds only), supporting the robustness of gene-level thresholds (Supplementary Table S12; Fig. 2).

While these findings support the use of gene-level thresholds for *in silico* evidence, many clinically actionable genes currently lack a sufficient number of classified variants for gene-level analysis. Given the NPV, PPV, and OPP for generalized thresholds are high (all >90%; Supplementary Table S5), and their prediction performance is superior to that of SIFT/PolyPhen2 agreement, generalized thresholds derived from the full set of 66 genes and their 90% CI are also presented (Supplementary Table S12). For REVEL, the 90% CI estimated for generalized thresholds for benign and deleterious evidence overlap with those estimated at the gene-level for 7/20 and 8/20 genes, respectively. For BayesDel, overlapping 90% CI for generalized and gene-level thresholds indicating benign or deleterious evidence were observed for 9/20 and 7/20 genes, respectively. We note that for REVEL, the generalized threshold’s 90% CI estimated for benign evidence overlaps with that estimated for gene-level pathogenic evidence for 2 genes (*MYBPC3* and *NSD1*); likewise, the 90% CI around the generalized threshold estimated for pathogenic evidence overlapped with that estimated for gene-level benign evidence for 1 gene (*TSC2*). No overlap between the generalized threshold’s 90% CI for benign evidence and CIs estimated for gene-level pathogenic evidence was observed for any genes using BayesDel, however, the 90% CI around the generalized threshold for pathogenic evidence overlapped with that for gene-level benign evidence for *TSC2*.

## Discussion

In this study, we evaluated and compared the performance of 5 well known meta-predictors often used for *in silico* assessment in variant classification. Our results were consistent with studies that found meta-predictors better assessed variant pathogenicity than concordance of individual predictors^2,4–6^. Our findings suggest that REVEL and BayesDel outperform the other 3 meta-predictors (Fig. 1). We also observed superior performance of gene-level thresholds compared to generalized thresholds. When evaluated on a gene-by-gene basis, REVEL and BayesDel achieved the highest OPP. In addition, sensitivity analysis in a subset of missense variants unlikely to have been included in the training datasets of the meta-predictors yielded similar results.

The strengths of our approach include generation of gene-level thresholds for variant assessment in 20 clinically actionable genes, and comparison to generalized thresholds estimated from a broader set of 66 such genes. Both gene-level and generalized thresholds yielded NPV, PPV and overall performance statistics ≥90%, and whether estimated from REVEL or BayesDel, both outperformed SIFT/PolyPhen2 agreement. However, our results also demonstrated improved prediction performance when assigning *in silico* evidence using gene-level thresholds compared to generalized thresholds, which highlights the importance of gene-specific assessment of variant pathogenicity^21^. We further observed wide variation in gene-level thresholds, and that the 90% CI around the generalized thresholds overlapped with those of the gene-level thresholds for <50% of the 20 genes examined in this study. Taken together, our findings support the use of gene-level thresholds for clinical variant assessment, when available.

An additional strength of our approach is the use of 2-sided thresholds for evidence assignment, as opposed to the current binary thresholds recommended for meta-predictors such as REVEL and BayesDel^2^. The use of 2-sided thresholds for evidence assignment is a data-driven solution to the problem of uncertainty; a single threshold necessarily assigns evidence to all variants but at the cost of high false positive or false negative rates. However, we acknowledge the trade-off between accuracy and yield, as thresholds quantified from Firth logistic regression models may be overly conservative in protecting against false predictions at the expense of the proportion of variants for which benign or pathogenic evidence can be assigned. Future methods development may include non-linear models or a weighted mechanism for adaptive balance between accuracy and yield designed to improve the overall performance of evidence assignment.

Our findings are based on the use of ClinVar-curated variants, a rapidly growing database with confidently annotated disease-causing alterations, as a training set to calibrate *in silico* scores on a gene-by-gene basis. In view of potential circularity issues arising from the testing of variants available in ClinVar that may have been previously used to train the meta-predictors we analyzed in the present study, we performed a sensitivity analysis to assess the impact of these variants. However, we were precluded from fully accounting for all such variants due to the fact that some of the variants included in our sensitivity analysis may have been deposited in ClinVar prior to 2015 and/or included in HGMD or ExAC at the time the models were developed. Nonetheless, our sensitivity analysis results were consistent with those of the larger analysis based on the full set of variants.

## Conclusions

Assigning *in silico* evidence using gene-level 2-sided thresholds from REVEL or BayesDel scores achieved higher predictive accuracy and yield compared to the use of other *in silico* predictors. The REVEL and BayesDel thresholds we report can serve as a viable resource for assigning *in silico* evidence to missense variants in the genes examined herein, to be used in conjunction with other lines of evidence in variant assessment as recommended in ACMG/AMP guidelines.

## Supplementary information

Supplementary Information

Supplementary Dataset

## Data Availability

All the data necessary to produce the results of this article is included in Supplementary data.
