# Supplementary material for: REVEL and BayesDel outperform other in silico meta-predictors for clinical variant classification
Source: Sci Rep. 2019 Sep 4;9:12752. doi: 10.1038/s41598-019-49224-8 (PMC6726608; doi:10.1038/s41598-019-49224-8)
Supplement: Supplementary file 1 — Supplementary Information [file 41598_2019_49224_MOESM1_ESM.docx]

**Supplementary Information**

**REVEL and BayesDel outperform other *in silico* meta-predictors for clinical variant classification**

Yuan Tian, Tina Pesaran, Adam Chamberlin, R. Bryn Fenwick, Shuwei Li, Chia-Ling Gau, Elizabeth C. Chao, Hsiao-Mei Lu, Mary Helen Black, Dajun Qian

**Table S1.** *In silico* predictors. **Table S2.** Number of classified missense variants by gene. **Table S3.** Number of missing values of *in silico* scores by gene. **Table S4.** Comparison of evidence assignment using REVEL versus BayesDel (2,153 variants in 20 genes). **Table S5.** Prediction performance of *in silico* evidence assignment (4,094 variants in 66 genes). **Table S6.** Comparison of evidence assignment using REVEL versus BayesDel (4,094 variants in 66 genes). **Table S7.** Comparison of evidence assignment using REVEL versus SIFT/ PolyPhen2 agreement (2,153 variants in 20 genes). **Table S8**. Comparison of evidence assignment using BayesDel versus SIFT/PolyPhen2 agreement (2,153 variants in 20 genes). **Table S9.** Comparison of evidence assignment using REVEL versus SIFT/PolyPhen2 agreement (4,094 variants in 66 genes). **Table S10.** Comparison of evidence assignment using BayesDel versus SIFT/PolyPhen2 agreement (4,094 variants in 66 genes). **Table S11.** Sensitivity analysis of prediction performance of *in silico* evidence in all variants in 20 genes versus subsets reported after the training of meta-predictors. **Table S12.** Thresholds for assigning benign and deleterious *in silico* evidence in missense variants (DOCX, 109 kb).

**Supplementary data**. ClinVar-classified missense variants in 66 genes (n = 4,094) (XLSX, 724 kb)

**Table S1.** *In silico* predictors

| Predictor | Description^a^ |
| --- | --- |
| SIFT | SIFT[^1^](#_ENREF_1) predicts whether a single amino acid substitution affects protein function based on the conservation of amino acid residues in sequence alignments derived from closely related sequences. The scores range from 0 to 1 with a smaller value representing a stronger damaging effect. The binary threshold for deleterious is <0.05. Website: http://sift.jcvi.org/ |
| PolyPhen2 | PolyPhen2[^2^](#_ENREF_2) predicts possible impact of an amino acid substitution on the structure and function of a human protein using straightforward physical and comparative considerations. The scores from HVAR data are used and range from 0 to 1. The evidence assessment uses 2 categories of possibly/probably damaging versus benign, or score >0.8 for deleterious. Website: http://genetics.bwh.harvard.edu/pph2/ |
| CADD | CADD[^3^](#_ENREF_3) score integrates multiple annotations into one metric by contrasting variants that survived natural selection with simulated mutations. Allele frequency is not used in the estimation. The scores range from 0 to 99. The binary threshold for deleterious is >19. Website: http://cadd.gs.washington.edu/ |
| MetaSVM | MetaSVM[^4^](#_ENREF_4) employs support vector machine algorithm to integrate 9 prediction scores (SIFT, PolyPhen2, GERP++, MutationTaster, MutationAssessor, FATHMM, LRT, SiPhy and PhyloP) and maximum minor allele frequency for evaluation of deleteriousness of missense mutations. The scores range from -2 to 3. The binary threshold for deleterious is >0.5. Website: https://omictools.com/meta-svm-tool |
| Eigen | Eigen[^5^](#_ENREF_5) is an unsupervised learning algorithm that combines a range of predictors to form clusters of functional and non-functional nucleotide positions in the genome. The scores using full annotation covariance matrix (i.e., Eigen, not Eigen_PC) are used. Allele frequency is used in the estimation. The scores range from -3 to 2. The binary threshold for deleterious is >0. Website: https://omictools.com/eigen-tool |
| REVEL | REVEL[^6^](#_ENREF_6) is an ensemble score for predicting the pathogenicity of missense variants based on a combination of 13 individual scores: MutPred, FATHMM v2.3, VEST 3.0, Polyphen-2, SIFT, PROVEAN, MutationAssessor, MutationTaster, LRT, GERP++, SiPhy, phyloP, and phastCons. Allele frequency is not used in the estimation. The scores range from 0 to 1. The binary threshold for deleterious is >0.5. Website: https://sites.google.com/site/revelgenomics/ |
| BayesDel | BayesDel[^7^](#_ENREF_7) is a combined deleteriousness score that optimize a weighted product of likelihood ratios of presumably correlated predictors. The scores without using allele frequency information (i.e., BayesDel_noAF, not BayesDel_useAF) are used. The scores range from -1.1 to 0.9. The binary threshold for deleterious is >0. Website: http://fengbj-laboratory.org/perch-2018-03-07/Home.html |

^a^The binary threshold for deleterious versus benign evidence of each *in silico* predictor is based on either author-recommended or inferred cut-offs. The *in silico* predictors are listed in order of publication date.

**Table S2.** Number of classified missense variants by gene

| Gene by group^a^ | B/LB^b^ | P/LP^b^ | Total |
| --- | --- | --- | --- |
| 20 genes in group A: |  |  |  |
| *ATM* | 50 | 31 | 81 |
| *ATP7B* | 10 | 12 | 22 |
| *BRCA1* | 206 | 63 | 269 |
| *BRCA2* | 346 | 39 | 385 |
| *CFTR* | 12 | 58 | 70 |
| *COL3A1* | 20 | 21 | 41 |
| *FBN1* | 15 | 139 | 154 |
| *KCNH2* | 15 | 138 | 153 |
| *MLH1* | 36 | 89 | 125 |
| *MSH2* | 30 | 52 | 82 |
| *MSH6* | 35 | 16 | 51 |
| *MUTYH* | 10 | 18 | 28 |
| *MYBPC3* | 28 | 72 | 100 |
| *NF1* | 16 | 19 | 35 |
| *NSD1* | 16 | 10 | 26 |
| *RET* | 22 | 33 | 55 |
| *RYR2* | 21 | 90 | 111 |
| *SCN5A* | 31 | 112 | 143 |
| *TP53* | 15 | 88 | 103 |
| *TSC2* | 100 | 19 | 119 |
| 46 genes in group B: |  |  |  |
| *ACVRL1* | 0 | 10 | 10 |
| *AKAP9* | 54 | 0 | 54 |
| *ANK2* | 28 | 4 | 32 |
| *ANKRD11* | 0 | 1 | 1 |
| *APC* | 45 | 5 | 50 |
| *APOB* | 10 | 2 | 12 |
| *BARD1* | 19 | 0 | 19 |
| *BRIP1* | 11 | 2 | 13 |
| *CDH1* | 23 | 5 | 28 |
| *CDKN2A* | 6 | 15 | 21 |
| *CHD7* | 13 | 2 | 15 |
| *DHCR7* | 2 | 15 | 17 |
| *DMD* | 57 | 5 | 62 |
| *DNAAF1* | 7 | 0 | 7 |
| *DNAH11* | 40 | 0 | 40 |
| *DNAH5* | 16 | 2 | 18 |
| *DSG2* | 26 | 7 | 33 |
| *DSP* | 32 | 7 | 39 |
| *ENG* | 8 | 3 | 11 |
| *FBN2* | 28 | 8 | 36 |
| *FH* | 3 | 42 | 45 |
| *FLNA* | 20 | 4 | 24 |
| *GBA* | 6 | 22 | 28 |
| *GCK* | 0 | 6 | 6 |
| *KCNQ1* | 7 | 139 | 146 |
| *LDLR* | 2 | 24 | 26 |
| *LMNA* | 0 | 51 | 51 |
| *MEN1* | 3 | 23 | 26 |
| *MYH7* | 9 | 200 | 209 |
| *MYLK* | 12 | 0 | 12 |
| *NBN* | 14 | 0 | 14 |
| *NOTCH1* | 20 | 0 | 20 |
| *PALB2* | 22 | 0 | 22 |
| *PMS2* | 34 | 4 | 38 |
| *POLE* | 42 | 0 | 42 |
| *PTCH1* | 28 | 1 | 29 |
| *PTEN* | 0 | 58 | 58 |
| *PTPN11* | 1 | 93 | 94 |
| *SDHB* | 6 | 21 | 27 |
| *STK11* | 2 | 14 | 16 |
| *TNNT2* | 3 | 35 | 38 |
| *TSC1* | 38 | 1 | 39 |
| *TTN* | 344 | 4 | 348 |
| *TTR* | 5 | 14 | 19 |
| *VHL* | 2 | 24 | 26 |
| *VPS13B* | 19 | 1 | 20 |
| Total | 2,101 | 1,993 | 4,094 |

^a^Each of 20 genes in group A contained at least 10 B/LB and 10 P/LP classified variants; each of 46 genes in group B contained fewer than 10 B/LB or 10 P/LP classified variants.

^b^The numbers of B/LB and P/LP variants were categorized based on ClinVar consensus classifications. B/LB, benign or likely benign variants; P/LP, pathogenic or likely pathogenic variants.

**Table S3.** Number of missing values of *in silico* scores by gene^a^

| Gene | SIFT | Polyphen2 | CADD | MetaSVM | Eigen | REVEL | BayesDel |
| --- | --- | --- | --- | --- | --- | --- | --- |
| *ACVRL1* (n = 10) | 0 | 0 | 0 | 0 | 0 | 0 | 0 |
| *AKAP9* (n = 54) | 0 | 0 | 0 | 0 | 0 | 0 | 0 |
| *ANK2* (n = 32) | 0 | 0 | 0 | 0 | 0 | 0 | 0 |
| *ANKRD11* (n = 1) | 0 | 0 | 0 | 0 | 0 | 0 | 0 |
| *APC* (n = 50) | 0 | 0 | 0 | 0 | 0 | 0 | 0 |
| *APOB* (n = 12) | 0 | 0 | 0 | 0 | 0 | 0 | 0 |
| *ATM* (n = 81) | 0 | 0 | 0 | 0 | 0 | 0 | 0 |
| *ATP7B* (n = 22) | 0 | 0 | 0 | 0 | 0 | 0 | 0 |
| *BARD1* (n = 19) | 0 | 0 | 0 | 0 | 0 | 0 | 0 |
| *BRCA1* (n = 269) | 0 | 0 | 0 | 1 (0.4%) | 0 | 0 | 0 |
| *BRCA2* (n = 385) | 0 | 0 | 0 | 0 | 0 | 0 | 0 |
| *BRIP1* (n = 13) | 0 | 0 | 0 | 0 | 0 | 0 | 0 |
| *CDH1* (n = 28) | 0 | 0 | 0 | 0 | 0 | 0 | 0 |
| *CDKN2A* (n = 21) | 0 | 0 | 0 | 1 (4.8%) | 0 | 0 | 0 |
| *CFTR* (n = 70) | 0 | 0 | 0 | 0 | 0 | 0 | 0 |
| *CHD7* (n = 15) | 0 | 0 | 0 | 0 | 0 | 0 | 0 |
| *COL3A1* (n = 41) | 0 | 0 | 0 | 0 | 0 | 0 | 0 |
| *DHCR7* (n = 17) | 0 | 0 | 0 | 0 | 0 | 0 | 0 |
| *DMD* (n = 62) | 0 | 0 | 0 | 0 | 62 (100.0%) | 0 | 0 |
| *DNAAF1* (n = 7) | 0 | 0 | 0 | 0 | 0 | 0 | 0 |
| *DNAH11* (n = 40) | 0 | 23 (57.5%) | 0 | 0 | 0 | 0 | 0 |
| *DNAH5* (n = 18) | 0 | 0 | 0 | 0 | 0 | 0 | 0 |
| *DSG2* (n = 33) | 0 | 0 | 0 | 0 | 0 | 0 | 0 |
| *DSP* (n = 39) | 0 | 0 | 0 | 0 | 0 | 0 | 0 |
| *ENG* (n = 11) | 0 | 0 | 0 | 0 | 0 | 0 | 0 |
| *FBN1* (n = 154) | 0 | 0 | 0 | 0 | 0 | 0 | 0 |
| *FBN2* (n = 36) | 0 | 0 | 0 | 0 | 0 | 0 | 0 |
| *FH* (n = 45) | 0 | 0 | 0 | 0 | 0 | 0 | 0 |
| *FLNA* (n = 24) | 0 | 0 | 0 | 0 | 24 (100.0%) | 0 | 0 |
| *GBA* (n = 28) | 0 | 0 | 0 | 0 | 0 | 0 | 0 |
| *GCK* (n = 6) | 0 | 0 | 0 | 0 | 0 | 0 | 0 |
| *KCNH2* (n = 153) | 0 | 0 | 0 | 0 | 0 | 0 | 0 |
| *KCNQ1* (n = 146) | 0 | 0 | 0 | 0 | 0 | 0 | 0 |
| *LDLR* (n = 26) | 0 | 0 | 0 | 0 | 0 | 0 | 0 |
| *LMNA* (n = 51) | 0 | 0 | 0 | 0 | 0 | 0 | 0 |
| *MEN1* (n = 26) | 0 | 0 | 0 | 0 | 0 | 0 | 0 |
| *MLH1* (n = 125) | 0 | 0 | 0 | 0 | 0 | 0 | 0 |
| *MSH2* (n = 82) | 0 | 0 | 0 | 0 | 0 | 0 | 0 |
| *MSH6* (n = 51) | 0 | 0 | 0 | 0 | 0 | 0 | 0 |
| *MUTYH* (n = 28) | 0 | 0 | 0 | 1 (3.6%) | 0 | 0 | 0 |
| *MYBPC3* (n = 100) | 0 | 0 | 0 | 0 | 0 | 0 | 0 |
| *MYH7* (n = 209) | 0 | 0 | 0 | 0 | 0 | 0 | 0 |
| *MYLK* (n = 12) | 0 | 0 | 0 | 0 | 0 | 0 | 0 |
| *NBN* (n = 14) | 0 | 0 | 0 | 0 | 0 | 0 | 0 |
| *NF1* (n = 35) | 0 | 0 | 0 | 0 | 0 | 0 | 0 |
| *NOTCH1* (n = 20) | 0 | 0 | 0 | 0 | 0 | 0 | 0 |
| *NSD1* (n = 26) | 0 | 0 | 0 | 0 | 0 | 0 | 0 |
| *PALB2* (n = 22) | 0 | 0 | 0 | 0 | 0 | 0 | 0 |
| *PMS2* (n = 38) | 0 | 0 | 0 | 0 | 0 | 0 | 0 |
| *POLE* (n = 42) | 0 | 0 | 0 | 0 | 0 | 0 | 0 |
| *PTCH1* (n = 29) | 0 | 0 | 0 | 0 | 0 | 0 | 0 |
| *PTEN* (n = 58) | 0 | 0 | 0 | 0 | 0 | 0 | 0 |
| *PTPN11* (n = 94) | 0 | 0 | 0 | 0 | 0 | 0 | 0 |
| *RET* (n = 55) | 0 | 0 | 0 | 0 | 0 | 0 | 0 |
| *RYR2* (n = 111) | 0 | 0 | 0 | 0 | 0 | 0 | 0 |
| *SCN5A* (n = 143) | 0 | 0 | 0 | 0 | 0 | 0 | 0 |
| *SDHB* (n = 27) | 0 | 0 | 0 | 0 | 0 | 0 | 0 |
| *STK11* (n = 16) | 0 | 0 | 0 | 0 | 0 | 0 | 0 |
| *TNNT2* (n = 38) | 0 | 0 | 0 | 0 | 0 | 0 | 0 |
| *TP53* (n = 103) | 0 | 0 | 0 | 0 | 0 | 0 | 0 |
| *TSC1* (n = 39) | 0 | 0 | 0 | 0 | 0 | 0 | 0 |
| *TSC2* (n = 119) | 0 | 0 | 0 | 0 | 0 | 0 | 0 |
| *TTN* (n = 348) | 209 (60.1%) | 0 | 0 | 0 | 0 | 0 | 0 |
| *TTR* (n = 19) | 0 | 0 | 0 | 0 | 0 | 0 | 0 |
| *VHL* (n = 26) | 0 | 0 | 0 | 0 | 0 | 0 | 0 |
| *VPS13B* (n = 20) | 0 | 0 | 0 | 0 | 0 | 0 | 0 |
| Total (n = 4,094) | 209 (5.1%) | 23 (0.6%) | 0 | 3 (0.1%) | 86 (2.1%) | 0 | 0 |

^a^ No. (%) of missing values by gene were summarized for 4,094 classified variants in 66 genes.

**Table S4.** Comparison of evidence assignment using REVEL versus BayesDel (2,153 variants in 20 genes)

| Cross validation outcomes^a^ | |  |
| --- | --- | --- |
| REVEL | BayesDel | No. (%) |
| Concordant evidence: 1,861 (86.4%) | |  |
| TP | TP | 789 (36.6%) |
| TN | TN | 740 (34.4%) |
| FP | FP | 25 (1.2%) |
| FN | FN | 24 (1.1%) |
| NE | NE | 283 (13.1%) |
| Favoring REVEL: 142 (6.6%) | |  |
| TP | FN | 0 |
| TN | FP | 1 (0.1%) |
| TP | NE | 69 (3.2%) |
| TN | NE | 43 (2.0%) |
| NE | FP | 13 (0.6%) |
| NE | FN | 16 (0.7%) |
| Favoring BayesDel: 150 (7.0%) | |  |
| FN | TP | 2 (0.1%) |
| FP | TN | 0 |
| NE | TP | 68 (3.2%) |
| NE | TN | 58 (2.7%) |
| FP | NE | 15 (0.7%) |
| FN | NE | 7 (0.3%) |

^a^Evidence assignment by REVEL and BayesDel were each assessed using gene-level thresholds by leave-one-out cross validation. TP, true positive; TN, true negative; FP, false positive; FN, false negative; NE, no evidence.

**Table S5.** Prediction performance of *in silico* evidence assignment (4,094 variants in 66 genes)

|  | Performance statistic (Rank)^a^ | | | | | | | | |
| --- | --- | --- | --- | --- | --- | --- | --- | --- | --- |
| Method | TP | TN | FP | FN | NE | PPV | NPV | YR | OPP |
| SIFT/PolyPhen2 agreement | 1,546 | 1,178 | 276 | 117 | 977 | 0.849 (11) | 0.910 (11) | 0.761 (7) | 0.842 (7) |
| Gene-level & generalized thresholds |  |  |  |  |  |  |  |  |  |
| CADD | 1,140 | 1,210 | 127 | 56 | 1,561 | 0.900 (7) | 0.956 (5) | 0.619 (9) | 0.838 (9) |
| MetaSVM | 1,552 | 1,595 | 85 | 72 | 790 | 0.948 (4) | 0.957 (4) | 0.807 (5) | 0.907 (3) |
| Eigen | 1,207 | 1,265 | 166 | 51 | 1,405 | 0.879 (8) | 0.961 (3) | 0.657 (8) | 0.842 (7) |
| REVEL | 1,546 | 1,685 | 56 | 60 | 747 | 0.965 (1) | 0.966 (1) | 0.818 (2) | 0.919 (1) |
| BayesDel | 1,506 | 1,699 | 59 | 79 | 751 | 0.962 (2) | 0.956 (5) | 0.817 (3) | 0.914 (2) |
| Generalized thresholds alone |  |  |  |  |  |  |  |  |  |
| CADD | 913 | 1,080 | 137 | 42 | 1,922 | 0.870 (9) | 0.963 (2) | 0.531 (11) | 0.809 (11) |
| MetaSVM | 1,568 | 1,572 | 110 | 108 | 736 | 0.934 (6) | 0.936 (10) | 0.820 (1) | 0.898 (5) |
| Eigen | 925 | 1,185 | 157 | 54 | 1,773 | 0.855 (10) | 0.956 (5) | 0.567 (10) | 0.810 (10) |
| REVEL | 1,563 | 1,580 | 79 | 88 | 784 | 0.952 (3) | 0.947 (9) | 0.809 (4) | 0.905 (4) |
| BayesDel | 1,511 | 1,558 | 98 | 84 | 843 | 0.939 (5) | 0.949 (8) | 0.794 (6) | 0.897 (6) |

^a^All performance statistics, except those for SIFT/ PolyPhen2 agreement, were evaluated by leave-one-out cross-validation. Ranks in parentheses were the descending orders of performance statistics among comparison methods. Using gene-level and generalized thresholds, the OPP statistics were higher for REVEL than those of CADD, MetaSVM and Eigen (*p* < 0.0001, 0.11 and <0.0001, respectively, by Monte Carlo permutation tests), higher for BayesDel than those of CADD, MetaSVM and Eigen (*p* < 0.0001, 0.33 and <0.0001, respectively), and equivalent between REVEL and BayesDel (*p* = 0.53). TN, true negative; FN, false negative; TP, true positive; FP, false positive; NE, no evidence; PPV, positive predictive value; NPV, negative predictive value; YR, yield rate; OPP, overall prediction performance.

**Table S6.** Comparison of evidence assignment using REVEL versus BayesDel (4,094 variants in 66 genes)

| Cross validation outcomes^a^ | |  |
| --- | --- | --- |
| REVEL | BayesDel | No. (%) |
| Concordant evidence: 3,500 (85.5%) | |  |
| TP | TP | 1,387 (33.9%) |
| TN | TN | 1,583 (38.7%) |
| FP | FP | 35 (0.9%) |
| FN | FN | 39 (1.0%) |
| NE | NE | 456 (11.1%) |
| Favoring REVEL: 319 (7.8%) | |  |
| TP | FN | 3 (0.1%) |
| TN | FP | 3 (0.1%) |
| TP | NE | 156 (3.8%) |
| TN | NE | 99 (2.4%) |
| NE | FP | 21 (0.5%) |
| NE | FN | 37 (0.9%) |
| Favoring BayesDel: 275 (6.7%) | |  |
| FN | TP | 2 (0.1%) |
| FP | TN | 0 |
| NE | TP | 117 (2.9%) |
| NE | TN | 116 (2.8%) |
| FP | NE | 21 (0.5%) |
| FN | NE | 19 (0.5%) |

^a^Evidence assignment by REVEL and BayesDel were each assessed using gene-level thresholds in 20 genes and generalized thresholds in 46 genes by leave-one-out cross validation. TP, true positive; TN, true negative; FP, false positive; FN, false negative; NE, no evidence.

**Table S7.** Comparison of evidence assignment using REVEL versus SIFT/ PolyPhen2 agreement (2,153 variants in 20 genes)

| Cross validation outcomes^a^ | |  |
| --- | --- | --- |
| REVEL | SIFT & PolyPhen2 | No. (%) |
| Concordant evidence: 1,463 (68.0%) | |  |
| TP | TP | 749 (34.8%) |
| TN | TN | 559 (26.0%) |
| FP | FP | 26 (1.2%) |
| FN | FN | 15 (0.7%) |
| NE | NE | 114 (5.3%) |
| Favoring REVEL: 439 (20.4%) | |  |
| TP | FN | 8 (0.4%) |
| TN | FP | 77 (3.6%) |
| TP | NE | 101 (4.7%) |
| TN | NE | 148 (6.9%) |
| NE | FP | 74 (3.4%) |
| NE | FN | 31 (1.4%) |
| Favoring SIFT & PolyPhen2: 251 (11.7%) | | |
| FN | TP | 5 (0.2%) |
| FP | TN | 5 (0.2%) |
| NE | TP | 134 (6.2%) |
| NE | TN | 85 (3.9%) |
| FP | NE | 9 (0.4%) |
| FN | NE | 13 (0.6%) |

^a^Evidence assignment by REVEL was assessed using gene-level thresholds by leave-one-out cross validation. Evidence of SIFT/PolyPhen2 agreement was assessed as deleterious if SIFT < 0.05 and possibly/probably damaging from PolyPhen2, or benign if SIFT ≥ 0.05 and benign from PolyPhen2. TP, true positive; TN, true negative; FP, false positive; FN, false negative; NE, no evidence.

**Table S8.** Comparison of evidence assignment using BayesDel versus SIFT/PolyPhen2 agreement (2,153 variants in 20 genes)

| Cross validation outcomes^a^ | |  |
| --- | --- | --- |
| BayesDel | SIFT & PolyPhen2 | No. (%) |
| Concordant evidence: 1,457 (67.7%) | |  |
| TP | TP | 746 (34.6%) |
| TN | TN | 565 (26.2%) |
| FP | FP | 25 (1.2%) |
| FN | FN | 13 (0.6%) |
| NE | NE | 108 (5.0%) |
| Favoring BayesDel: 444 (20.6%) | |  |
| TP | FN | 12 (0.6%) |
| TN | FP | 83 (3.9%) |
| TP | NE | 101 (4.7%) |
| TN | NE | 150 (7.0%) |
| NE | FP | 69 (3.2%) |
| NE | FN | 29 (1.3%) |
| Favoring SIFT & PolyPhen2: 252 (11.7%) | | |
| FN | TP | 9 (0.4%) |
| FP | TN | 6 (0.3%) |
| NE | TP | 133 (6.2%) |
| NE | TN | 78 (3.6%) |
| FP | NE | 8 (0.4%) |
| FN | NE | 18 (0.8%) |

^a^Evidence of BayesDel was assessed using gene-level thresholds by leave-one-out cross validation. Evidence of SIFT/PolyPhen2 agreement was assessed as deleterious if SIFT < 0.05 and possibly/probably damaging from PolyPhen2, or benign if SIFT ≥ 0.05 and benign from PolyPhen2. TP, true positive; TN, true negative; FP, false positive; FN, false negative; NE, no evidence.

**Table S9.** Comparison of evidence assignment using REVEL versus SIFT/PolyPhen2 agreement (4,094 variants in 66 genes)

| Cross validation outcomes^a^ | |  |
| --- | --- | --- |
| REVEL | SIFT & PolyPhen2 | No. (%) |
| Concordant evidence: 2,679 (65.4%) | |  |
| TP | TP | 1,338 (32.7%) |
| TN | TN | 1,045 (25.5%) |
| FP | FP | 33 (0.8%) |
| FN | FN | 23 (0.6%) |
| NE | NE | 240 (5.9%) |
| Favoring REVEL: 1,031 (25.2%) | |  |
| TP | FN | 20 (0.5%) |
| TN | FP | 134 (3.3%) |
| TP | NE | 188 (4.6%) |
| TN | NE | 506 (12.4%) |
| NE | FP | 109 (2.7%) |
| NE | FN | 74 (1.8%) |
| Favoring SIFT & PolyPhen2: 384 (9.4%) | | |
| FN | TP | 12 (0.3%) |
| FP | TN | 5 (0.1%) |
| NE | TP | 196 (4.8%) |
| NE | TN | 128 (3.1%) |
| FP | NE | 18 (0.4%) |
| FN | NE | 25 (0.6%) |

^a^Evidence of REVEL was assessed using gene-level thresholds in 20 genes and generalized thresholds in 46 genes by leave-one-out cross validation. Evidence of SIFT/PolyPhen2 agreement was assessed as deleterious if SIFT < 0.05 and possibly/probably damaging from PolyPhen2, or benign if SIFT ≥ 0.05 and benign from PolyPhen2. TP, true positive; TN, true negative; FP, false positive; FN, false negative; NE, no evidence.

**Table S10.** Comparison of evidence assignment using BayesDel versus SIFT/PolyPhen2 agreement (4,094 variants in 66 genes)

| Cross validation outcomes^a^ | |  |
| --- | --- | --- |
| BayesDel | SIFT & PolyPhen2 | No. (%) |
| Concordant evidence: 2,613 (63.8%) | |  |
| TP | TP | 1,287 (31.4%) |
| TN | TN | 1,043 (25.5%) |
| FP | FP | 33 (0.8%) |
| FN | FN | 19 (0.5%) |
| NE | NE | 231 (5.6%) |
| Favoring BayesDel: 1,037 (25.3%) | |  |
| TP | FN | 40 (1.0%) |
| TN | FP | 139 (3.4%) |
| TP | NE | 179 (4.4%) |
| TN | NE | 517 (12.6%) |
| NE | FP | 104 (2.5%) |
| NE | FN | 58 (1.4%) |
| Favoring SIFT & PolyPhen2: 444 (10.8%) | | |
| FN | TP | 26 (0.6%) |
| FP | TN | 10 (0.2%) |
| NE | TP | 233 (5.7%) |
| NE | TN | 125 (3.1%) |
| FP | NE | 16 (0.4%) |
| FN | NE | 34 (0.8%) |

^a^Evidence of BayesDel was assessed using gene-level thresholds in 20 genes and generalized thresholds in 46 genes by leave-one-out cross validation. Evidence of SIFT/PolyPhen2 agreement was assessed as deleterious if SIFT < 0.05 and possibly/probably damaging from PolyPhen2, or benign if SIFT ≥ 0.05 and benign from PolyPhen2. TP, true positive; TN, true negative; FP, false positive; FN, false negative; NE, no evidence.

**Table S11.** Sensitivity analysis of prediction performance of *in silico* evidence in all variants in 20 genes versus subsets reported after the training of meta-predictors

|  | Performance statistic^b^ | | | | | | | | |
| --- | --- | --- | --- | --- | --- | --- | --- | --- | --- |
| Method^a^ | TP | TN | FP | FN | NE | PPV | NPV | YR | OPP |
| Full dataset (2,153 variants in 20 genes) |  |  |  |  |  |  |  |  |  |
| CADD | 746 | 707 | 60 | 38 | 602 | 0.926 | 0.949 | 0.720 | 0.871 |
| MetaSVM | 848 | 746 | 57 | 39 | 463 | 0.937 | 0.950 | 0.785 | 0.894 |
| Eigen | 850 | 761 | 51 | 27 | 464 | 0.943 | 0.966 | 0.784 | 0.901 |
| REVEL | 858 | 784 | 40 | 33 | 438 | 0.955 | 0.960 | 0.797 | 0.907 |
| BayesDel | 859 | 798 | 39 | 40 | 417 | 0.957 | 0.952 | 0.806 | 0.908 |
| Sub dataset (870 variants in 20 genes) |  |  |  |  |  |  |  |  |  |
| CADD | 278 | 272 | 30 | 14 | 276 | 0.903 | 0.951 | 0.683 | 0.853 |
| MetaSVM | 331 | 286 | 33 | 14 | 206 | 0.909 | 0.953 | 0.763 | 0.879 |
| Eigen | 325 | 296 | 25 | 6 | 218 | 0.929 | 0.980 | 0.749 | 0.892 |
| REVEL | 347 | 303 | 22 | 13 | 185 | 0.940 | 0.959 | 0.787 | 0.899 |
| BayesDel | 351 | 310 | 22 | 16 | 171 | 0.941 | 0.951 | 0.803 | 0.901 |

^a^Full set included all missense variants with ClinVar consensus classifications in 20 genes. Sub dataset contained variants in which each variant had submissions only after September 2015 and its consensus classification was defined from those submitted to ClinVar during September 2015 to August 2017.

^b^All performance statistics were evaluated by leave-one-out cross-validation method. TP, true positive; TN, true negative; FP, false positive; FN, false negative; NE, no evidence; PPV, positive predictive value; NPV, negative predictive value; YR, yield rate; OPP, overall prediction performance.

**Table S12** Thresholds for assigning benign and deleterious *in silico* evidence in missense variants^a^

| By gene or in all | Thresholds of REVEL scores | | Thresholds of BayesDel scores | |
| --- | --- | --- | --- | --- |
| genes combined | T_BE_ (90% CI) | T_DE_ (90% CI) | T_BE_ (90% CI) | T_DE_ (90% CI) |
| *ATM* | 0.359 (0.291, 0.443) | 0.689 (0.600, 0.763) | -0.180 (-0.242, -0.110) | 0.216 (0.102, 0.314) |
| *ATP7B* | 0.514 (0.446, 0.590) | 0.731 (0.660, 0.784) | -0.076 (-0.167, 0.042) | 0.248 (0.181, 0.310) |
| *BRCA1* | 0.628 (0.601, 0.658) | 0.824 (0.784, 0.869) | 0.147 (0.115, 0.186) | 0.425 (0.382, 0.473) |
| *BRCA2* | 0.581 (0.554, 0.616) | 0.974 (0.900, 1.000) | 0.080 (0.043, 0.118) | 0.500 (0.418, 0.584) |
| *CFTR* | 0.438 (0.318, 0.556) | 0.727 (0.673, 0.769) | -0.032 (-0.130, 0.063) | 0.277 (0.225, 0.322) |
| *COL3A1* | 0.515 (0.431, 0.629) | 0.762 (0.685, 0.812) | 0.026 (-0.061, 0.170) | 0.329 (0.234, 0.393) |
| *FBN1* | 0.326 (0.231, 0.417) | 0.597 (0.549, 0.632) | -0.328 (-0.482, -0.198) | 0.047 (-0.008, 0.092) |
| *KCNH2* | 0.417 (0.365, 0.476) | 0.649 (0.612, 0.684) | -0.176 (-0.264, -0.086) | 0.127 (0.084, 0.162) |
| *MLH1* | 0.109 (0.000, 0.304) | 0.815 (0.758, 0.883) | 0.107 (0.011, 0.208) | 0.423 (0.394, 0.451) |
| *MSH2* | 0.562 (0.484, 0.648) | 0.862 (0.814, 0.903) | 0.085 (0.009, 0.183) | 0.426 (0.369, 0.475) |
| *MSH6* | 0.556 (0.483, 0.671) | 0.881 (0.765, 0.962) | 0.095 (0.020, 0.233) | 0.419 (0.310, 0.488) |
| *MUTYH* | 0.214 (0.059, 0.409) | 0.661 (0.511, 0.766) | -0.078 (-0.197, 0.103) | 0.263 (0.108, 0.357) |
| *MYBPC3* | 0.013 (0.000, 0.135) | 0.511 (0.441, 0.587) | -0.531 (-0.691, -0.409) | 0.012 (-0.059, 0.086) |
| *NF1* | 0.261 (0.159, 0.389) | 0.605 (0.487, 0.698) | -0.191 (-0.279, -0.074) | 0.077 (-0.006, 0.136) |
| *NSD1* | 0.400 (0.318, 0.504) | 0.705 (0.448, 0.772) | -0.082 (-0.189, 0.049) | 0.268 (0.164, 0.327) |
| *RET* | 0.481 (0.402, 0.580) | 0.732 (0.666, 0.785) | -0.122 (-0.246, 0.022) | 0.300 (0.203, 0.388) |
| *RYR2* | 0.349 (0.287, 0.426) | 0.597 (0.526, 0.649) | -0.233 (-0.304, -0.162) | 0.038 (-0.045, 0.095) |
| *SCN5A* | 0.425 (0.376, 0.487) | 0.704 (0.653, 0.745) | -0.108 (-0.163, -0.043) | 0.180 (0.121, 0.229) |
| *TP53* | 0.536 (0.490, 0.586) | 0.667 (0.620, 0.706) | -0.003 (-0.027, 0.030) | 0.132 (0.058, 0.183) |
| *TSC2* | 0.703 (0.662, 0.773) | 0.970 (0.893, 1.000) | 0.244 (0.194, 0.307) | 0.561 (0.478, 0.657) |
| Generalized thresholds | 0.460 (0.448, 0.472) | 0.741 (0.732, 0.750) | -0.074 (-0.086, -0.061) | 0.268 (0.256, 0.280) |

^a^The 2-sided thresholds T_BE_ and T_DE_ for assigning BE and DE were estimated at the predicted probabilities of pathogenicity 0.2 and 0.8, respectively, given all variants tested. 90% confidence intervals (90% CI) were each estimated for T_BE_ or T_DE_ in 10,000 bootstrapping replicates stratified by classification status B/LB vs. P/LP. The generalized thresholds were estimated from the combined set of 66 genes. BE, benign evidence; DE, deleterious evidence.

**Supplementary References**

1 Kumar, P., Henikoff, S. & Ng, P. C. Predicting the effects of coding non-synonymous variants on protein function using the SIFT algorithm. *Nat Protocols* **4**, 1073-1081 (2009).

2 Adzhubei, I. A. *et al.* A method and server for predicting damaging missense mutations. *Nat. Methods* **7**, 248-249 (2010).

3 Kircher, M. *et al.* A general framework for estimating the relative pathogenicity of human genetic variants. *Nat. Genet.* **46**, 310-315 (2014).

4 Dong, C. *et al.* Comparison and integration of deleteriousness prediction methods for nonsynonymous SNVs in whole exome sequencing studies. *Hum. Mol. Genet.* **24**, 2125-2137 (2015).

5 Ionita-Laza, I., McCallum, K., Xu, B. & Buxbaum, J. D. A spectral approach integrating functional genomic annotations for coding and noncoding variants. *Nat. Genet.* **48**, 214-220 (2016).

6 Ioannidis, N. M. *et al.* REVEL: An ensemble method for predicting the pathogenicity of rare missense variants. *Am. J. Hum. Genet.* **99**, 877-885 (2016).

7 Feng, B. J. PERCH: A unified framework for disease gene prioritization. *Hum. Mutat.* **38**, 243-251 (2017).
